# Supplementary material for: Unlocking the passivation nature of the cathode–air interfacial reactions in lithium ion batteries
Source: Nat Commun. 2020 Jun 25;11:3204. doi: 10.1038/s41467-020-17050-6 (PMC7316795; doi:10.1038/s41467-020-17050-6)
Supplement: Supplementary file 1 — Supplementary Information [file 41467_2020_17050_MOESM1_ESM.pdf]

Supplementary Information for

Unlocking the passivation nature of the cathode-air interfacial reaction in lithium ion batteries

Zou et al.

Supplementary Figures

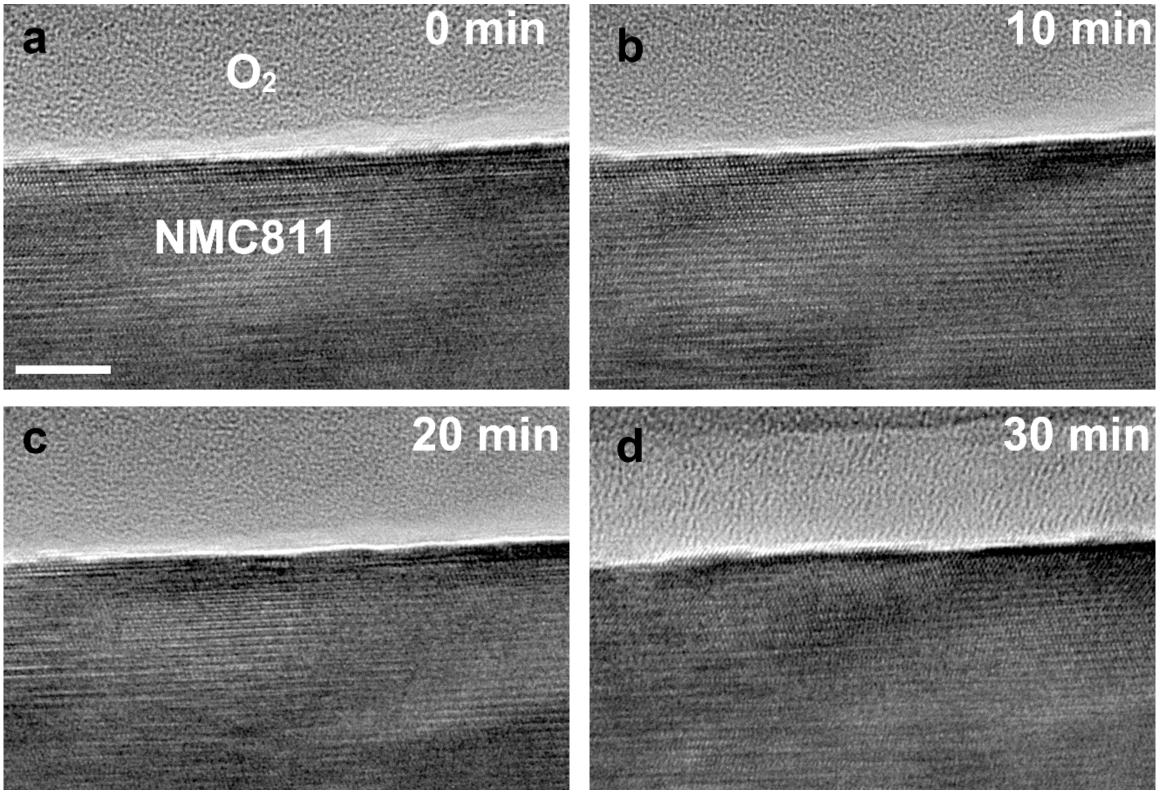

Supplementary Figure 1 Surface evolution of NMC811 in O_2 with a constant pressure of P_{O2}=5x10^{-2} Torr at room-temperature. (a-d) The surface morphology of NMC811 after the O_2 exposure of 0 min, 10 mins, 20 mins, and 30 mins, respectively. Scale bar, 5 nm **a**.

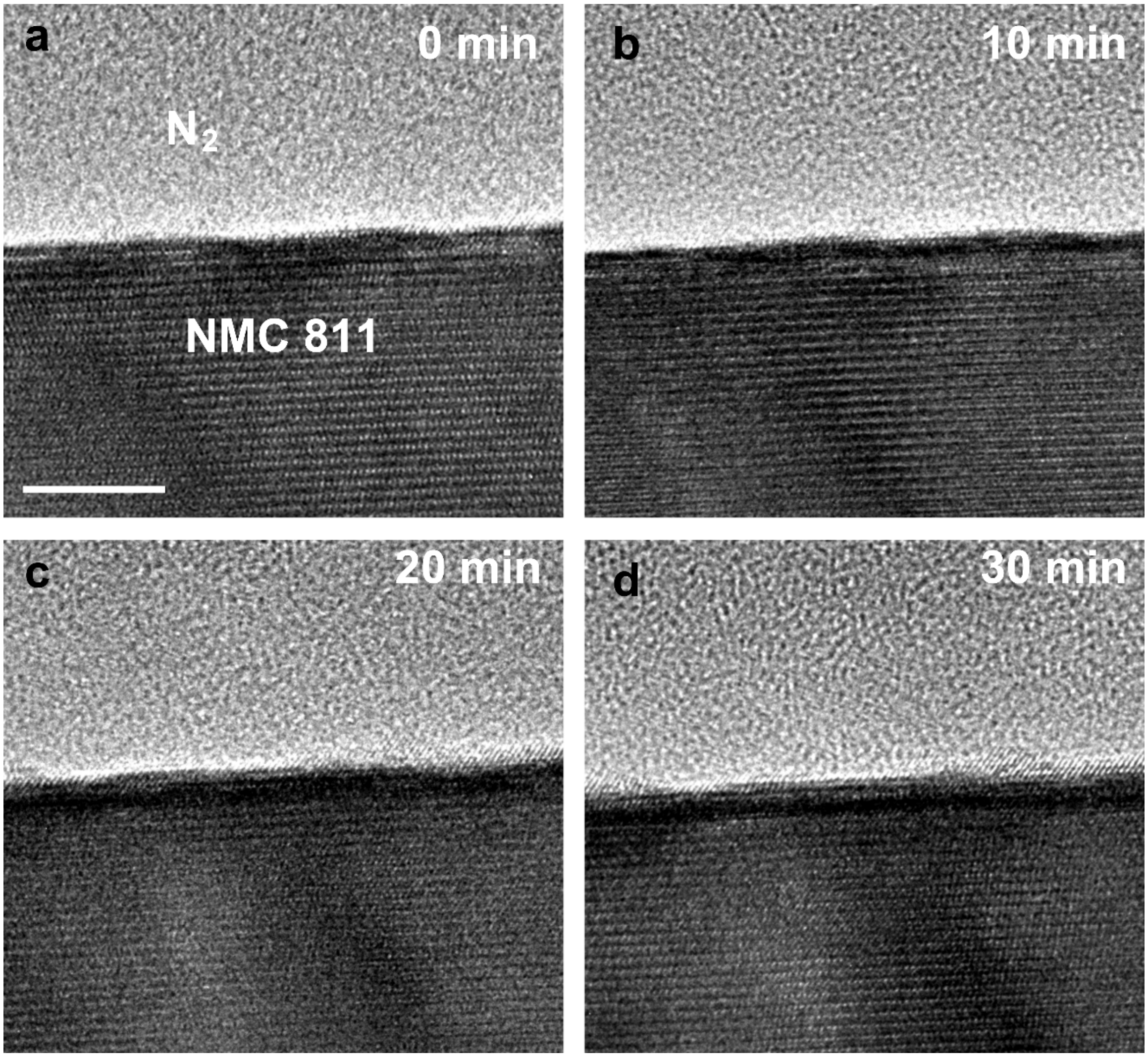

Supplementary Figure 2 Surface evolution of NMC811 in N_2 with a constant pressure of P_{N2}=5x10^{-2} Torr at room-temperature. (a-d) The surface morphology of NMC811 after the N_2 exposure of 0 min, 10 mins, 20 mins, and 30 mins, respectively. Scale bar, 5 nm **a**.

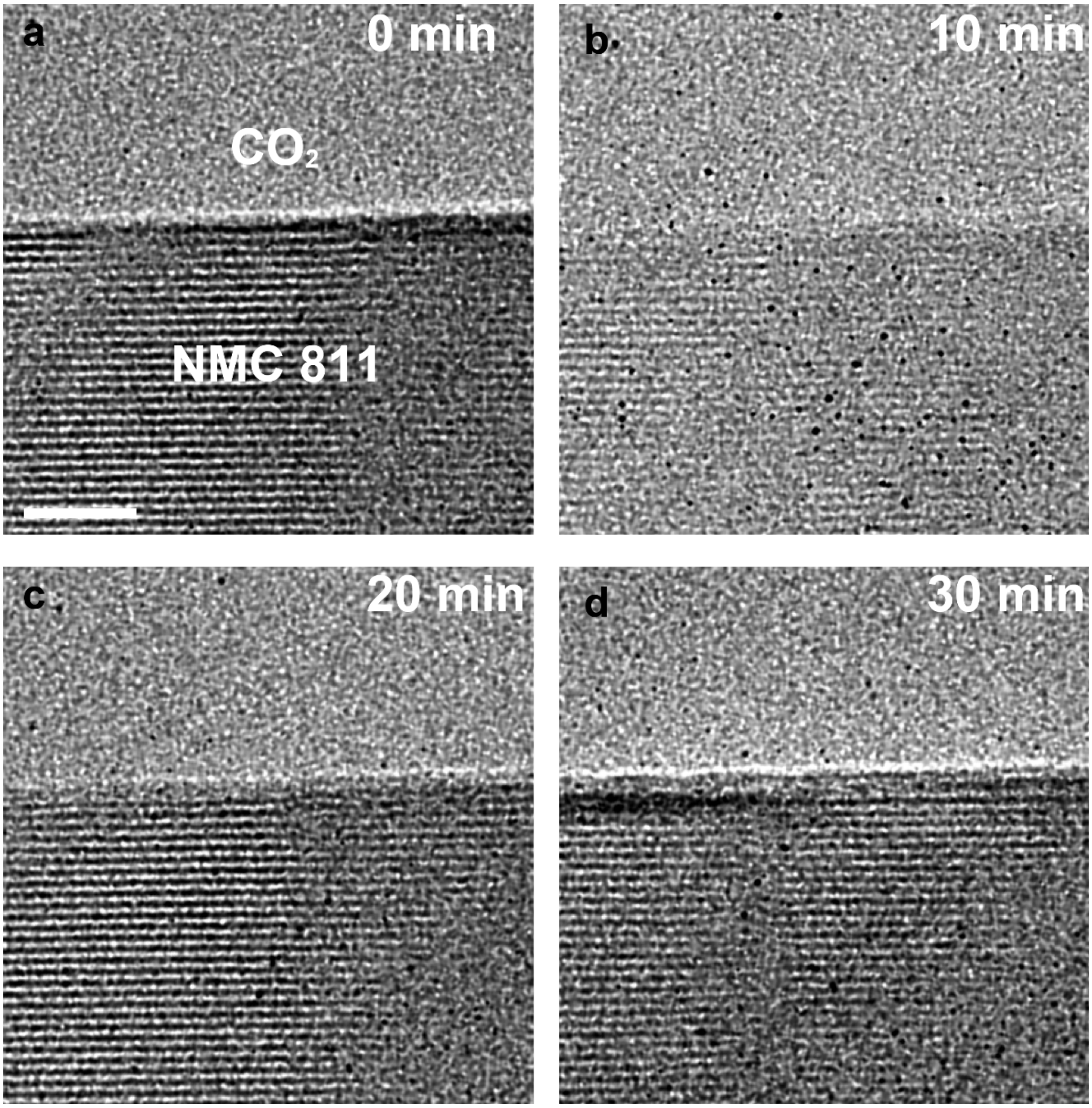

Supplementary Figure 3 Surface evolution of NMC811 in CO_2 with a constant pressure of P_{CO2}=5x10^{-2} Torr at room-temperature. (a-d) The surface morphology of NMC811 after the CO_2 exposure of 0 min, 10 mins, 20 mins, and 30 mins, respectively. Scale bar, 5 nm **a**.

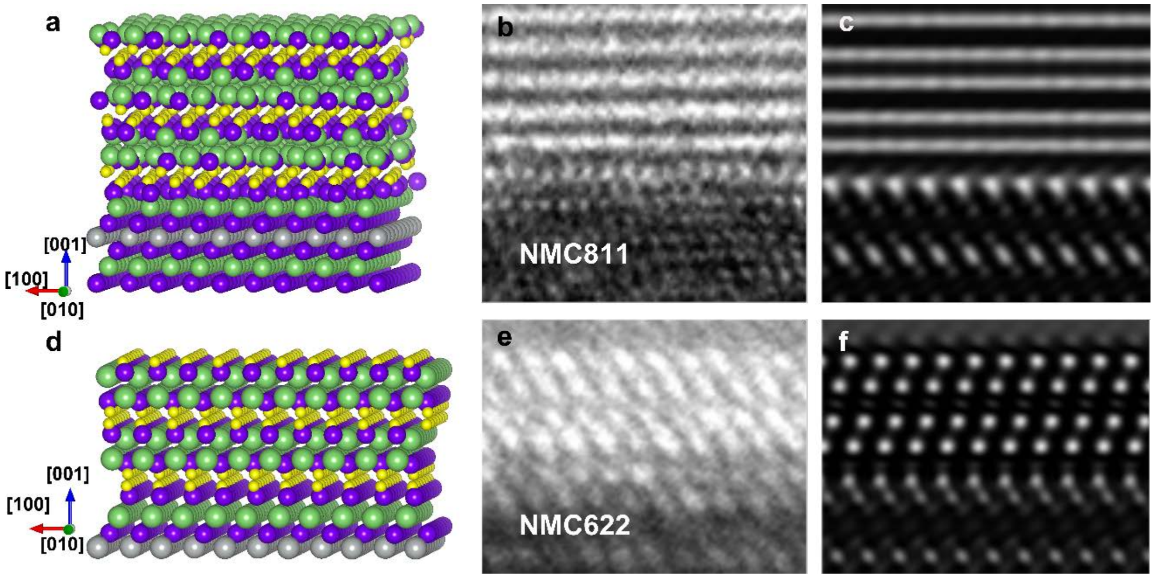

Supplementary Figure 4 Interfacial matching of LiOH and NMC derivatives. (a-c) Interface model, HRTEM image, and simulated contrast of LiOH/NMC811 interface with the orientation of LiOH(001)//NMC(001) and LiOH[230]//NMC[1-10]. **(d-f)** Interface model, HRTEM image, and simulated contrast of LiOH/NMC622 interface with the orientation of LiOH(001)//NMC(001) and LiOH[010]//NMC[110].

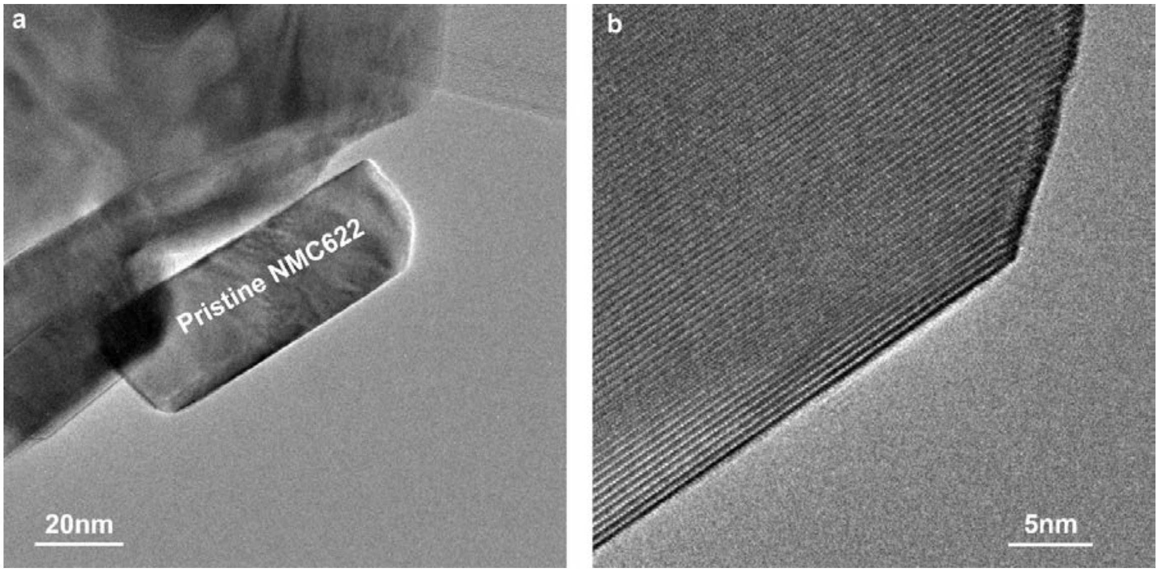

Supplementary Figure 5 HRTEM image showing the representative pristine sample used in the ETEM experiments. (a) TEM image of pristine NMC622. (b) Atomic scale view of the pristine NMC622 surface.

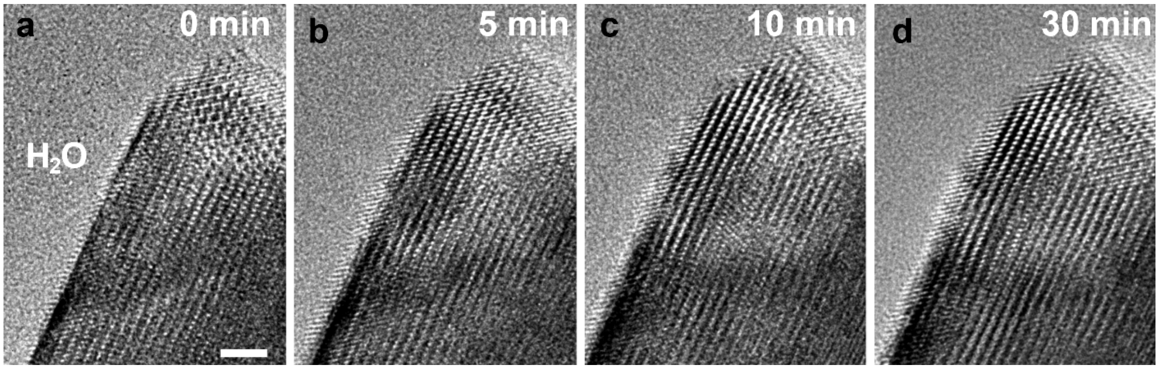

Supplementary Figure 6 Surface evolution of NMC333 in H_2O with a constant pressure of P_{H2O}=5x10^{-2} Torr at room-temperature. (a-d) The surface morphology of NMC333 after the H_2O exposure of 0 min, 5 mins, 10 mins, and 30 mins, respectively. Scale bar, 2 nm **a**.

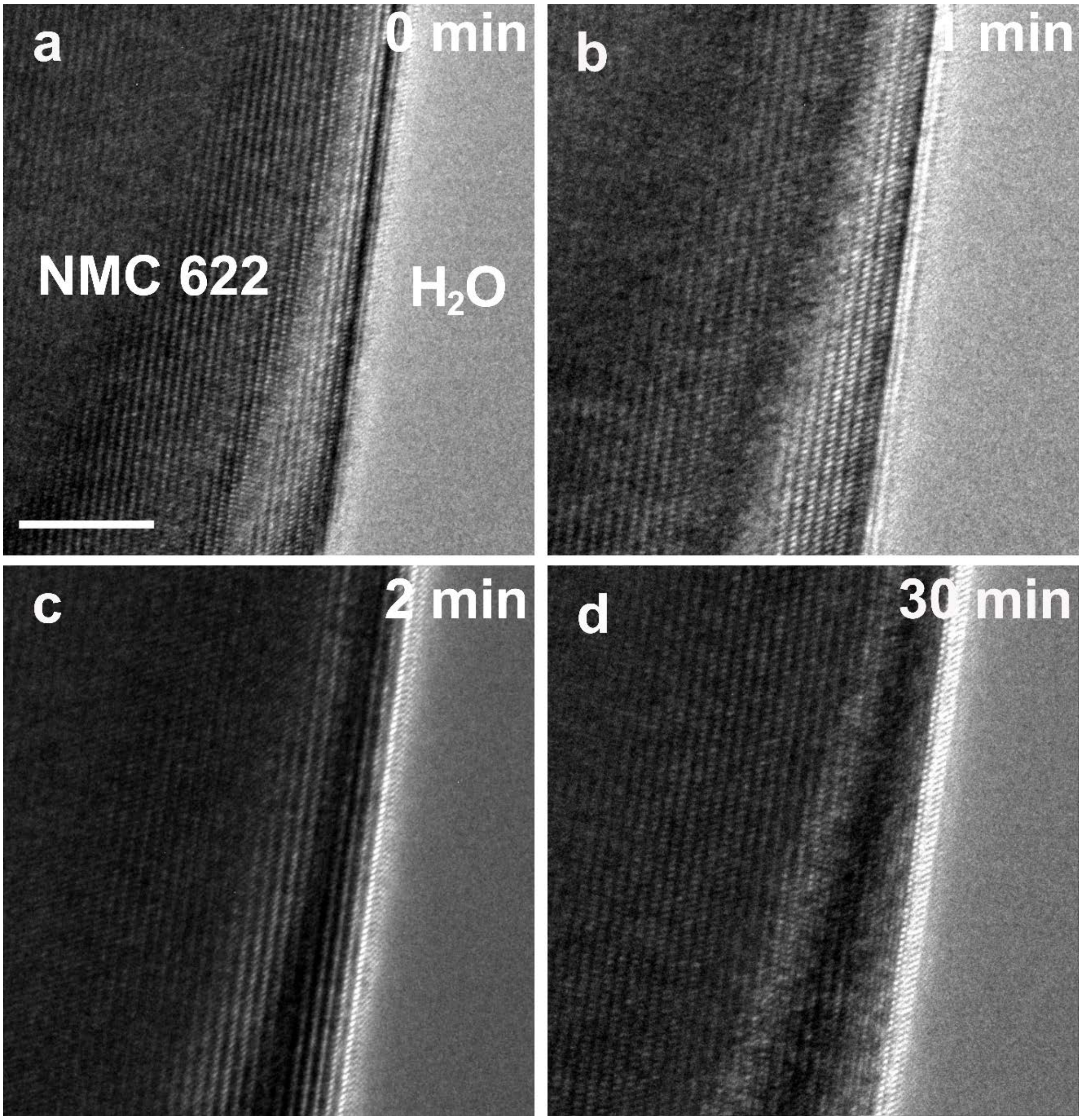

Supplementary Figure 7 Surface evolution of NMC622 in H_2O with a constant pressure of P_{H2O}=5x10^{-2} Torr at room-temperature. (a-d) The surface morphology of NMC622 after the H_2O exposure of 0 min, 1 mins, 2 mins, and 30 mins, respectively. Scale bar, 5 nm **a**.

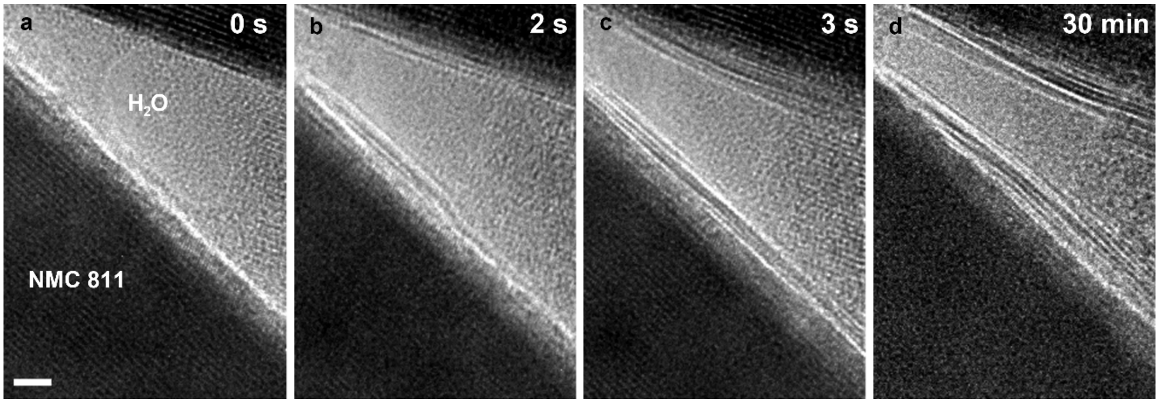

Supplementary Figure 8 Surface evolution of NMC811 in H_2O with a constant pressure of P_{H2O}=5x10^{-2} Torr at room-temperature. (a-d) The surface morphology of NMC622 after the H_2O exposure of 0s, 2s, 3s, and 30 mins, respectively. Scale bar, 3 nm **a**.

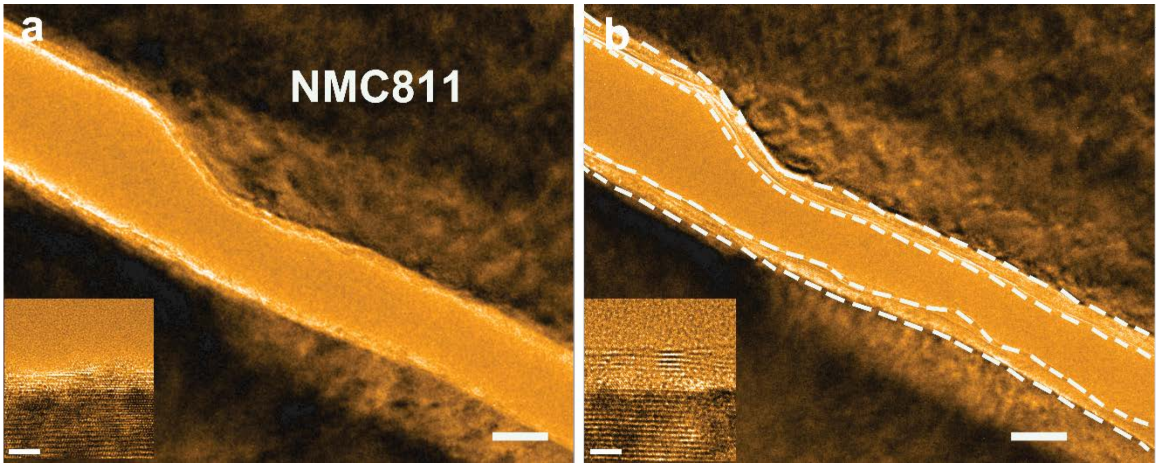

Supplementary Figure 9 (a) TEM image shows the surface structural feature of NMC811 prior to exposure to water vapor and the inset is an enlarged image of surface, showing no formation of LiOH. **(b)** TEM image of the same region in **b** after exposing to water vapor for 30 min, revealing the formation of a surface passivation layer of 6 atomic layers. The white dashed lines outline the boundary of hydroxide films and the insert displays the zoom in view of LiOH capped NMC811 surface. The blue dashed lines outline the boundary for the LiOH layers. Scale bar, 10 nm **a, b**. Scale bar, 3 nm inserts of **a, b**.

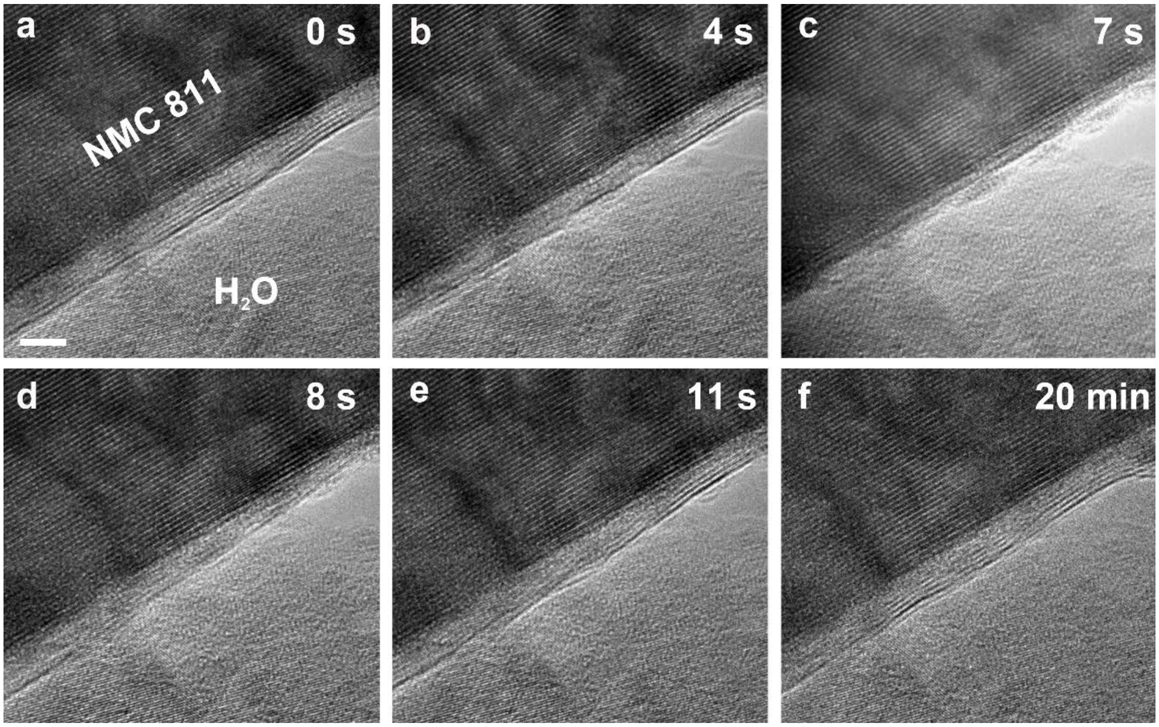

Supplementary Figure 10 Global view of self-healing of passivation layers. The sequences are captured under the continuous exposure to H_2O with a pressure of P_{H2O}= $5x10^{-2}$Torr and at room temperature. (a) NMC811 covered with a saturated hydroxide film. **(b-c)** The hydroxide thickness evolution as the condense of electron beam. **(d-f)** The hydroxide thickness evolution as the weakening of electron beam. Scale bar, 5 nm **a.**

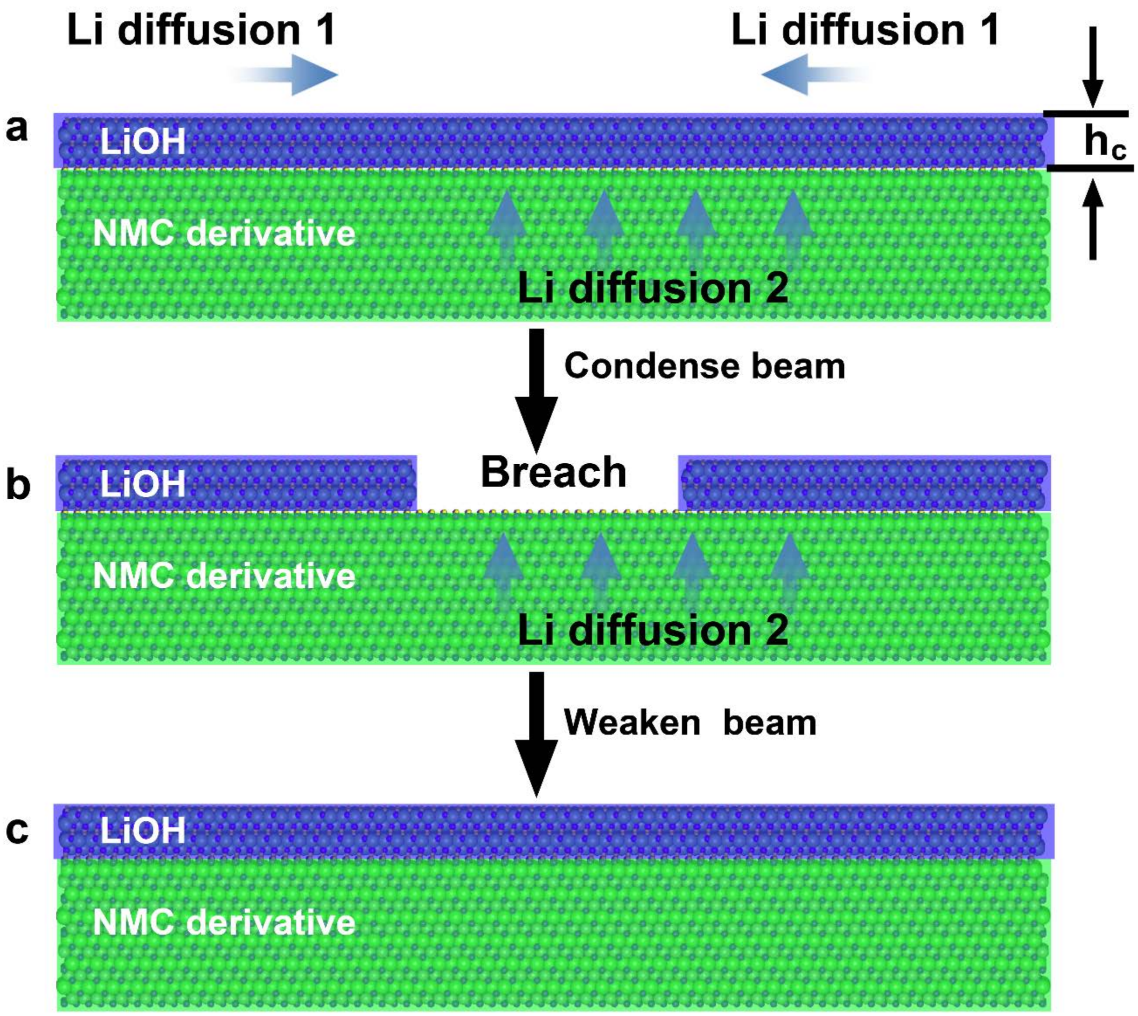

Supplementary Figure 11 Schematic drawing of the Li ions supply for the hydroxide growth. (a) Possible Li resources for the hydroxide growth. Path 1 and path 2 represent the Li ions supply from external source by surface diffusion, and the Li supply from NMC bulk through the bulk diffusion, respectively. **(b)** The creation of local breach by condensing the electron beam after reach the saturated thickness. **(c)** The self-healing of the breach by the supply of NMC bulk.

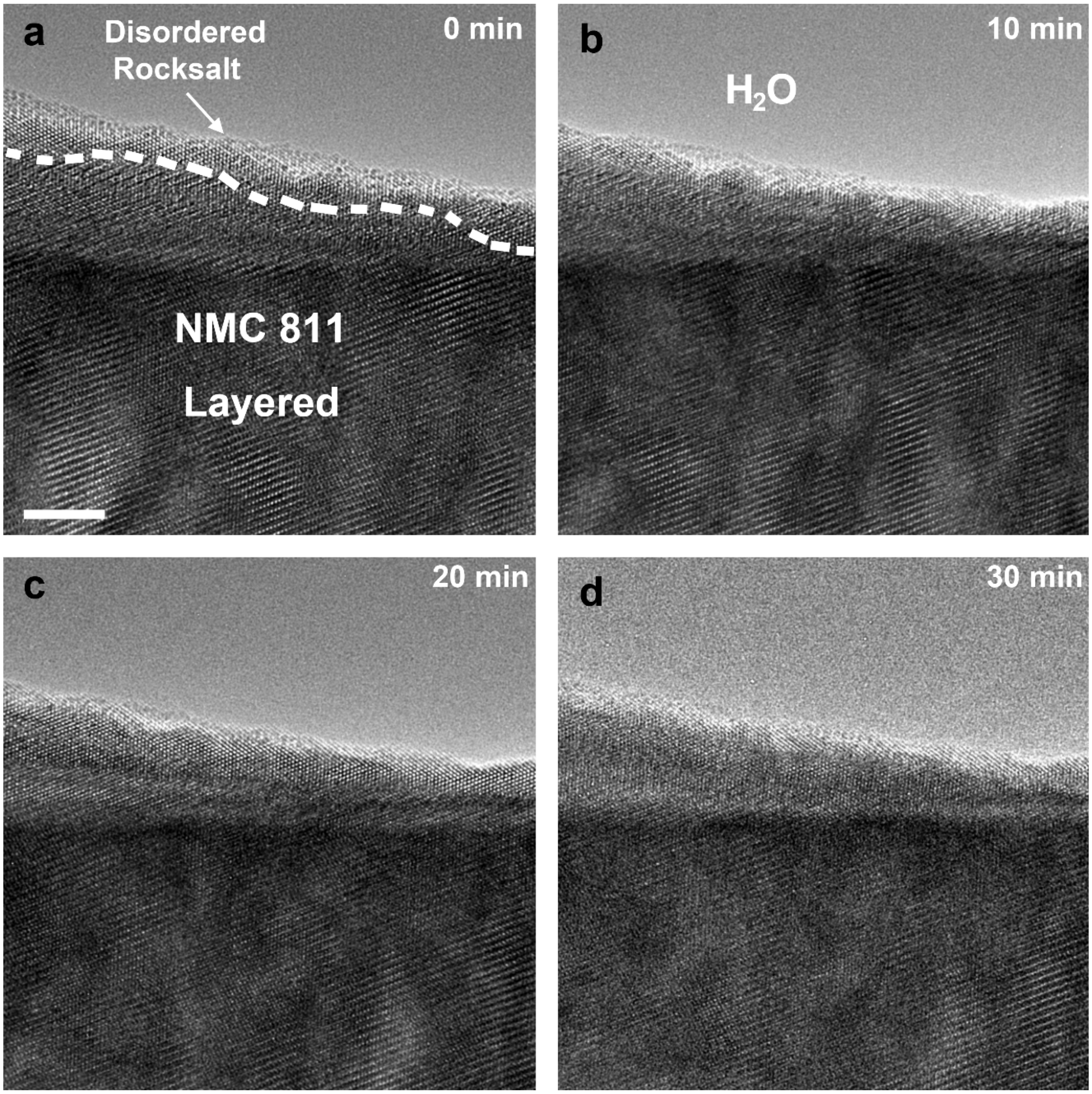

Supplementary Figure 12 Surface evolution of cycled NMC811 with a disordered rock salt shell in H_2O with a constant pressure of P_{H2O}=5x10^{-2} Torr at room-temperature. (a) The 10 cycled NMC811 with a reconstruction layer on surface. The white dashed lines outline the boundary between the layered core and the disordered rock salt shell. **(b-d)** The surface evolution of the core-shell NMC811 after the H_2O exposure of 10 mins, 20 mins, and 30 mins, respectively. Scale bar, 5 nm **a.**

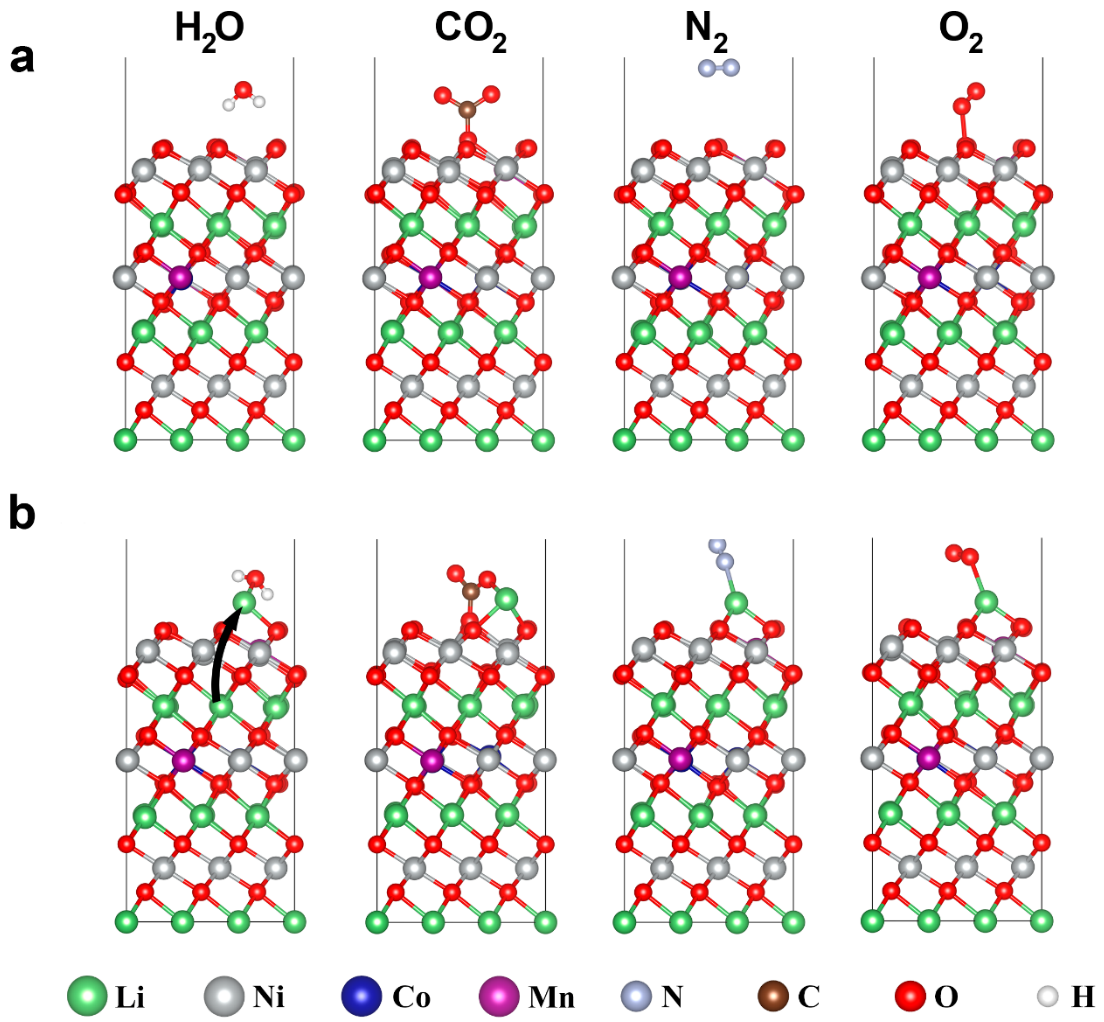

Supplementary Figure 13 Li surface segregation under an individual gas adsorption. **(a)** DFT relaxed NMC structure under the adsorption of H_2O, CO_2, N_2, and O_2. **(b)** DFT relaxed NMC structure with a Li moves from subsurface to surface 3a site.

Supplementary Notes

Supplementary Note 1 Interfaces of NMC derivates and LiOH

It is well-recognized that the reactions between Li and water results in the formation of LiOH . As the capped phase presents a good crystallinity, the entity of the reaction products can be further confirmed by the matching patterns at interfaces. The NMC and LiOH present two different types of interface alignment for NMC622/LiOH and NMC822/LiOH, in which the LiOH consistently present the one dimensional lattice while growing on the NMC811, whereas present the two dimensional features while growing on the NMC622. To verify the interfacial orientation between LiOH and NMC622/NMC811 phase, two different structural models are constructed to simulate the HRTEM contrast and compare with the obtained HRTEM images. A rotated model with the orientation of LiOH(001)//NMC(001) and LiOH[230]//NMC[1-10] is proposed for the LiOH/NMC811 interface, as shown in Supplementary Figure 4a. The simulated image reproduces the one dimensional lattice planes of the LiOH phase and the characteristic bright contrast of the oxygen layers (Supplementary Figure 4c), which qualitatively agrees with the HRTEM images (Supplementary Figure 4b). In a similar manner, a epitaixal model with the orientation of LiOH(001)//NMC(001) and LiOH[010]//NMC[110] is used to simulate the interface of LiOH/NMC622, as shown in Supplementary Figure 4d. The simulated image also generates the two dimensional lattice of LiOH phase as well as the characteristic bright contrast of oxygen columns (Supplementary Figure 4f), which agrees well with the HRTEM images (Supplementary Figure 4e). Notice that the real cathode samples are imperfect composed of binders and impurities, such features would affect the image contrast to some extend but are difficult to define in the atomistic models, therefore, the captured HRTEM images can show some deviations from the simulated contrast based on the perfect models, however, the characteristics of oxygen arrangements and lattice contrast are captured by the simulations, which confirms the formation of LiOH. The different interfacial alignment between LiOH and NMC derivatives may be associated with the growth kinetics and film thickness. On one hand, the LiOH demonstrates a much faster growth rate on the NMC811 substrate than that of NMC622, on the other hand, the LiOH possesses a larger saturated thickness, both of which can results in the distinct interfacial strain and serves as the driving force for developing distinct interfacial matching to reach the energy minimum.

Supplementary Note 2 Identify the Li ions supply for the LiOH growth

The pristine NMC samples are not clean with the presence of residual Li containing species on surfaces, therefore, it is essential to validate the effects of surface residue ions on the surface passivation behavior. As can be seen, there are two general Li ions sources that can fed the growth of hydroxide thin films: one is the residue Li ions from external resources, the other is the Li ions from the NMC compounds (supplementary Figure 11a). To support the local hydroxide films growth, the external Li ions have to arrive at the local site via the surface diffusion (path 1 in Supplementary Figure 11a), and the bulk Li ions have to percolate the thin films to come to the surface for reactions (path 2 in Supplementary Figure

11a). Once the hydroxide thin films growth stops, i.e., saturate at the crtical thickness (hc in Supplementary Figure 11a), the Li ions transportation are effectively impeded and no external Li ions are allowed to come to the local surface sites for reactions. With the removal of local LiOH passivation layers (Supplementary Figure 11b), the diffusion path 1 remains blocked, however, the barriers for the bulk Li ions transportation towards surface are removed. In this scenario, the surface breaches are immediatedly reparied (Supplementary Figure 11c), which indicates the bulk Li ions always serve as a ready supply for the growth of passivation layers. Meanwhile, in Fig.11a, both the surface and bulk can supply Li ions for the interfacial reactions, while in Fig.11c, only the bulk supply Li ions for the interfacial reactions, however, they are saturated at the same critical thickness. This clearly points to the fact that the surface residue Li does not alter the passivation nature of the interfacial reactions.
