# Supplementary material for: Unlocking the passivation nature of the cathode–air interfacial reactions in lithium ion batteries
Source: Nat Commun. 2020 Jun 25;11:3204. doi: 10.1038/s41467-020-17050-6 (PMC7316795; doi:10.1038/s41467-020-17050-6)
Supplement: Supplementary file 3 — Description of Additional Supplementary Information [file 41467_2020_17050_MOESM3_ESM.pdf]

Description of Additional Supplementary Files

File Name: Supplementary Movie 1
Description: Surface evolution of NMC333 in H2O.

File Name: Supplementary Movie 2
Description: Surface evolution of NMC622 in H2O.

File Name: Supplementary Movie 3
Description: Surface evolution of NMC811 in H2O.

File Name: Supplementary Movie 4
Description: Self-healing of passivation hydroxide layers.

File Name: Supplementary Movie 5
Description: Evolution of disordered rocksalt surface in H2O.
